# Answer to the comment on Castien et al. (2018) pressure pain thresholds over the cranio-cervical region in headache - a systematic review and meta-analysis

**DOI:** 10.1186/s10194-018-0858-y

**Published:** 2018-04-18

**Authors:** René F. Castien, Johannes C. van der Wouden, Willem De Hertogh

**Affiliations:** 1 Department of General Practice and Elderly Care Medicine, Amsterdam Public Health research institute, VU University Medical Center, van der Boechorststraat 7, Amsterdam, 1081 BT the Netherlands; 2 Healthcare center Haarlemmermeer, Waddenweg 1, Hoofddorp, 2134 XL the Netherlands; 3 Department of Rehabilitation Sciences and Physiotherapy, Faculty of Medicine and Health Sciences, University of Antwerp, Campus Drie Eiken, D.S.022, Universiteitsplein 1, Wilrijk, 2610 Belgium

With interest we have read Dr. Luedtke’s and colleagues comment regarding our paper: Pressure pain thresholds over the cranio-cervical region in headache - a systematic review and meta-analysis [1].

The authors mention a number of concerns they believe to affect our conclusions.

It appears that the authors believe the goal of our review is to elucidate the phenomenon of sensitization in patients with migraine, tension type headache and cervicogenic headache. This was however never our intention. As mentioned in the text, we aimed to present an overview of pressure pain thresholds (PPTs) in three different types of headache which are regularly treated by physical treatments that are focussed on the cranio-cervical region. We explicitly state that reduced PPTs ‘reflect signs of sensitization’ and ‘are supposed to reflect signs of sensitization’. We believe this careful formulation is sufficiently conditional to indicate we do not aim to assess presence or absence of sensitization with respect to headache. In our review, we restricted our focus on a frequently used, easy to administer, and reliable tool and outcome measure: pressure pain threshold in kg/cm^2^. The resulting discussion indeed indicates that this is a research area where further clarification is needed.

The difference between ictal and interictal measurements in migraine is important. As acknowledged by Luedtke et al., this is not always reported in the study reports and is therefore difficult to take into account. It is noteworthy that in Table 3, except Grossi 2011, all studies on PPT measured interictally at the midpoint of the trapezius muscle show a significant difference between migraine and controls. Overall, we are confident that the lack of some data concerning ictal-interictal measurements do not affect our conclusions.

The time lag between our search date and publication is due to the unavoidable delay caused by the entire manuscript preparation, submission and review process. Some of these are out of our control. By providing the time interval for searching studies we aimed to be transparent regarding possible new publications after our search date.

In the initial PROSPERO protocol, the Cochrane database was indeed mentioned as fourth database. We later decided to omit this database because the Cochrane database is primarily suited for research questions on effectiveness of interventions. We are confident that our search in Pubmed, Embase and Cinahl is sufficient. The three articles that are mentioned by the authors as missing articles were not excluded as a result of the omission of the Cochrane database. The simple reason is that they did not match the inclusion criteria of PPT measurement in the cranio-cervical region: the first concerns high-density EEG brain mapping, the second concerns measurements on the temporal muscle and in the third measurements over the supra-orbital nerve were performed.

To construct a comprehensive table with an overview of all main characteristics of the studies is a challenge. We regret the authors are unhappy with the result. Unfortunately, the data on gender (F/M) of Ashina and Fernandez de las Penas in the pdf-version are incorrectly placed in the column for blinding. We regret this typographical errors but are confident they will not distract the reader.

Risk of bias assessment was performed by using the Dutch EBRO checklist for case-control studies. The risk of bias tool developed by the Cochrane Collaboration is not suitable for this study design.

The last remark concerns the difference in number of included studies as stated in the abstract (*n* = 22) and in the main text (*n* = 17). Indeed, we failed to update the abstract. We apologize for this error. Luedtke et al. suggest that we may have changed the Prospero protocol retrospectively or may be reluctant to publish the registration number. In the free accessible PROSPERO protocol (ID=CRD42016033534) the authors could have noticed that the versions are identical and have not been changed.

Finally, we agree with Luedtke et al. that a systematic review on reporting the absence or presence of sensitization in different types of headache will be a valuable contribution to the literature.

René F. CastienJohannes C. van der WoudenWillem de Hertogh.
